# Perspectives of general practitioners towards their supervisors over the past ten years in China

**DOI:** 10.1186/s12909-022-03442-3

**Published:** 2022-05-31

**Authors:** Rao xin, Luo Li, Su Qiaoli, Wang Xingyue, Li Shuangqing

**Affiliations:** 1 Department of General practice, West China Hospital, SCU, Chengdu, China; 2 Institute of Hospital Management, West China Hospital, SCU, Chengdu, China; 3 Institute of Service Management, School of business, SCU, Chengdu, China; 4 Department of Graduate medical education, West China Hospital/School of Medcine, SCU, Chengdu, China

**Keywords:** GP Residents, GP Theoretical training supervisors, GP Hospital-based training supervisors, GP Community-based training supervisors

## Abstract

**Objectives:**

Doctors who wish to become general practitioners (GPs) in China are required to attend and complete general practice resident training. In the early stages of the standardized GP training system, GP training supervisors play important roles. This study aims to explore how GP residents perceive their GP supervisors, factors that affect GP residents’ satisfaction level, as well as to offer suggestions based on the implications of this study.

**Design:**

We conducted a mixed quantitative and qualitative study. Firstly, with respect to the quantitative research, we conducted a survey to investigate training satisfaction through questionnaires, then extracted and analyzed the factors that influence training satisfaction. In the qualitative study, we conducted in-depth, semistructured interviews using qualitative research criteria (COREQ)––a 32-item checklist for interviews.

**Participants:**

Participants in the quantitative survey included 1172 GPs whose training time wasbetween 2008 and 2017. Afterwards, 100 participants were selected from this sample , filtered by stratified random sampling and by having provided extreme answers on the quantitative survey (less than 5% of the total sample). They were chosen for the qualitative research to conduct a more detailed investigation., This stratified random sampling was based on residents’ grades, regions, and training levels (city level or county level). Extreme answers were identified as answers of “extremely good” or “extremely bad” to questions from the initial comprehensive survey of 1172 participants. Consequently, 30 participants with extreme answers were found, while the remaining 70 participants in the interviews were selected by stratified random sampling. Ultimately, this sample of participants met our information collection and sample estimation requirements.

**Results:**

The results show that satisfaction with GP theoretical training supervisors, GP hospital-based training supervisors, and GP community-based training supervisors differed. Considering long-term averages, the hierarchy of satisfaction is as follows: GP theoretical training supervisors > GP community-based training supervisors > GP hospital-based training supervisors.

GP hospital-based training supervisors need to improve their conception of GPs, teaching methods and conscientiousness. GP community-based training supervisors need to improve their teaching methods, knowledge of clinical theory and practice ability.

**Conclusions:**

On the one hand, teams of GP supervisors in China have gradually been established over time. On the other hand,the satisfaction tendencies of residents with respect to their GP supervisors are quite different, and teams of GP supervisors must be promoted and improved.

**Supplementary Information:**

The online version contains supplementary material available at 10.1186/s12909-022-03442-3.

## Background

Since reform of the health care system has become an important goal, general practice (GP) has been politically and practically improved in China [1, 2]. The national government established a target for human resources development in general practice, i.e., 2-3 ‘qualified GPs’ per 10,000 persons, and this figure is set to double by 2030 [3]. The Ministry of Health of China (MOH) regulates the training program for GP residents. This program includes ‘theoretical training’, ‘hospital-based training’ and ‘community clinic training’ [4]. The satisfaction of the GP residents program has been studied by many scholars [5–10], but how their attitudes towards their GP supervisors need further study. This study aims to explore how GP residents perceive their GP supervisors and the factors that affect their satisfaction levelsas well as to offer suggestions based on the implications of this study.

## Design

Mixed quantitative and qualitative approaches were applied.

### Quantitative study

At first, a quantitative survey was conducted to investigate GPs by collecting and analyzing their levels of satisfaction with their supervisors.

### Qualitative study

Then, in-depth, semi-structured interviews were conducted to complement the quantitative research and to investigate more deeply. Qualitative research criteria (COREQ) was used to assist the qualitative study design.

## Setting

This study was conducted by the research team, and participants completed the survey and the interview at their workplace at a convenient time.

## Participants

Between 2008 and 2017, a total of 1172 GP residents were enrolled in the training program in Shenzhen, China, and these individuals were received training at ten main GP training bases across different provinces in China (Eastern,Western and Middle of China). These training bases composed of the most important medical colleges and universities in the country and are representatives of the general practice training in China. A full sample population quantitative survey was conducted with these individuals as participants. The training program lasted for three years. In 2018, 532 participants had finished the program, and 640 participants were still in training. After the quantitative survey, qualitative research was conducted with 100 participants. For the qualitative research, 100 participants were investigateddeeper, and these participants were chosen either by stratified random sampling or by having provided extreme answers on the quantitative survey (less than 5% of the total sample).

### Questionnaire

Questionnaires were administered based on literatures and previous relevant practices. In July 2018, the questions used on these questionnaires were scrutinised by a group of experts and researchers consisting of two educational scientists, two GPs, and two professors. The feedback from these experts and researchers was used to adjust the questionnaires. Two rounds of feedback from the experts and researchers contributed to the improvement of the questionnaires.

### Independent variables

The first nine items in Table 1 were used as independent variables.

**Table 1:** Questionnaire concerning GP resident satisfaction

How satisfied are you with the following items (researchers were encouraged to express their opinions via the forced distribution method; a 6-point LIKERT scale was used, where a score of 1 was lowest and a score of 6 was highest; 1-3 indicated “unsatisfied”, while 4-6 indicated “satisfied”).							
1	The theoretical training process	1□	2□	3□	4□	5□	6□
2	The teaching skill of your theoretical supervisors	1□	2□	3□	4□	5□	6□
3	The GP clinic training process	1□	2□	3□	4□	5□	6□
4	Your hospital-based training supervisors	1□	2□	3□	4□	5□	6□
5	The GP community-based training process	1□	2□	3□	4□	5□	6□
6	Your community-based training supervisors	1□	2□	3□	4□	5□	6□
7	The training base infrastructure	1□	2□	3□	4□	5□	6□
8	The training organizational management	1□	2□	3□	4□	5□	6□
9	Your income during the training period	1□	2□	3□	4□	5□	6□
10	Your overall satisfaction	1□	2□	3□	4□	5□	6□

### Dependent variables

Overall satisfaction was used as the dependent variable.

A questionnaire was conducted to investigate aspects of GP training for supervisors that could be improved, as shown in Table 2.

**Table 2:** Questionnaire regarding aspects of GP training for supervisors that could be improved

Option	Choice (multiple )
① GP concept	
② Theoretical knowledge and clinical competence	
③ Motivation and responsibility for training	
④ Training skills and teaching methods	
⑤ Reciprocal feedback channels	
⑥ Sufficient opportunities for independent practice	
⑦ Training experience	
⑧ Other	
⑨ No need to improve	

We conducted pre-surveys and interviews to adjust the final questionnaire and list of prompts for interview questions. The final questionnaire featured a Cronbach’s alpha coefficient of 0.90 and an expert validity of 0.97.

### Interviews

For qualitative analysis, we conducted face-to-face, semi-structured interviews with GP residents, GP supervisors, and certain agency managers. The interviews were audio-recorded and transcribed. List of prompts for interview questions is as following Table 3.

**Table 3:** List of prompt interview questions

1. How satisfied are you with your GP theoretical training supervisors? Are they helpful to you during your training experience?
2. How satisfied are you with your GP hospital-based training supervisors? Are they helpful to you during your training experience?
3. How satisfied are you with your GP community training supervisors? Are they helpful to you during your training experience?
4. How familiar are your GP hospital-based training supervisors with community health affairs?
5. What progress could be made by specialist supervisors during your training experience in the clinical training context?
6. Are your supervisors conscientious regarding their teaching work?
7. Are your supervisors responsible with respect to their teaching work?
8. Are your supervisors skilled at their teaching work?
9. Do you receive helpful feedback from your supervisors?
10. If possible, describe an unhelpful experience with your supervisors. Why did this experience occur?
11. Do you enjoy keeping in contact with your supervisors after finishing the training program?
12. What is your overall advice to the whole training management staff?

### Analysis

In this quantitative survey, we used descriptive statistical methods to analyze the data and the logistic regression method to analyze the factors influencing GPs’ satisfaction with their training supervisors. In the qualitative analysis, primary themes were identified and categorised by the coinvestigators.

## Results

The basic information of GP resident students is reported in Table 4.

**Table 4:** Basic information of GP residents

Items	Category	Number	Percentage
Basic information of GP resident students (completed training)			
Sex	Male	226	42.60%
	Female	306	57.40%
Age	26~30	23	4.20%
	31~35	112	20.90%
	36~40	148	27.90%
	40+	249	47.00%
Degree	Others	169	31.90%
	Bachelor’s	317	59.60%
	Master	42	7.70%
	MD	4	0.80%
Professional	Junior or other	325	61.30%
	Mid-level	133	24.90%
	Vice-senior level	64	11.90%
	Senior level	10	1.90%
Employer	Hospital	301	56.60%
	Community health center (CHC)	165	31.10%
	Other	65	12.30%
Subtotal		532	
Basic information of GP resident students (in training)			
Sex	Male	201	42.60%
	Female	439	57.40%
Age	26~30	607	4.20%
	31~35	33	20.90%
Subtotal		640	
Total		1172	

### Descriptive statistics

**Fig. 1:**
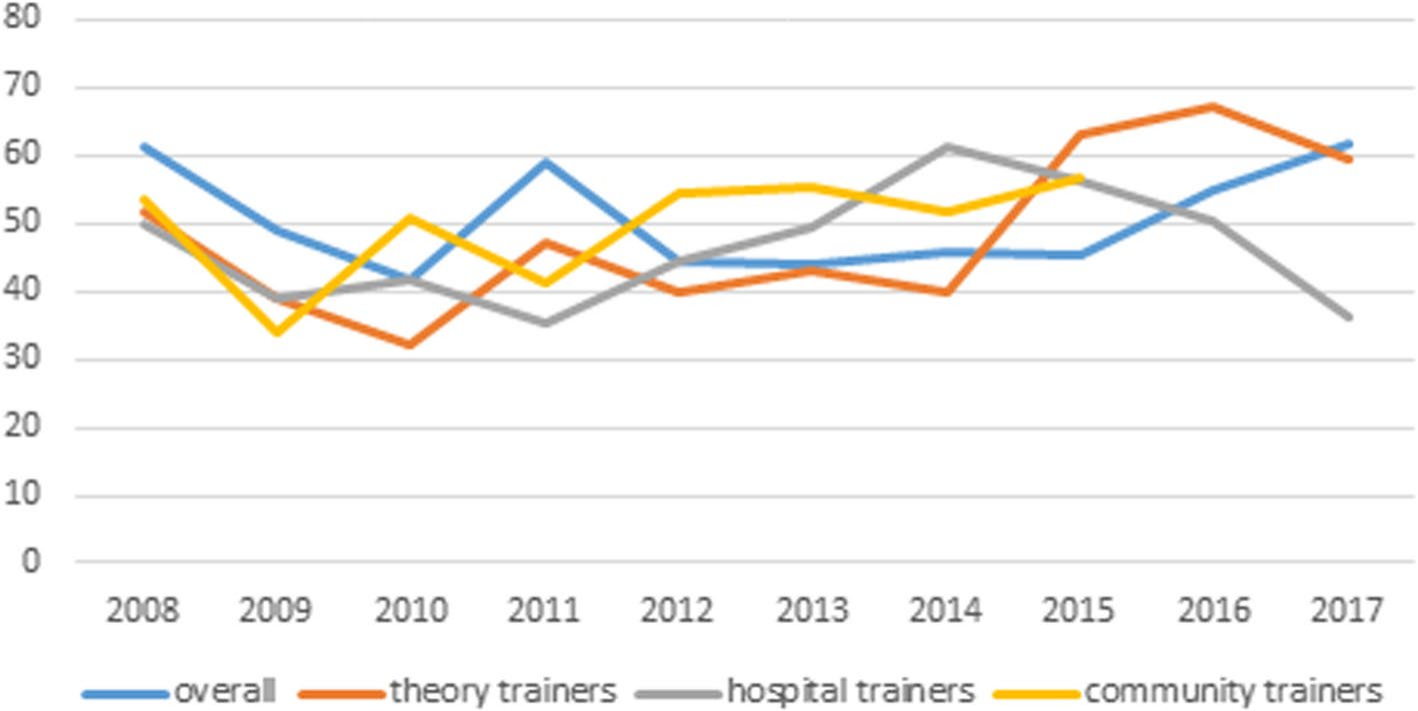
Trend map of GP satisfaction with GP supervisors

Figure 1 shows the trend map of GP satisfaction with GP supervisors (shown in supplemental materials).

### Data statistics

Figure 1 shows the ratio of “satisfied” to “unsatisfied”. As shown, overall satisfaction increases gradually and steadily. The results show that satisfaction with GP theoretical training supervisors grew rapidly from 2015 to 2017. Satisfaction with GP hospital-based training supervisors was relatively low at the early stage. Satisfaction rose gradually and steadily in general, but decreased during the periods 2008-2010 and 2015-2017. The quantitative study used the alternative hypothesis two-sided test, test power=0.90, alpha=0.05, significant coefficient *p*<0.05, indicating significant difference. The logistic regression showed that the higher participants’ satisfaction with organizational management (OR = 2.55), theoretical learning (OR = 2.63), theoretical curriculum supervisors (OR = 1.77), clinical base supervisors (OR = 2.56) and community practice teaching (OR = 1.87) was, the higher their overall satisfaction with GP training.

In hospital clinical rotations, supervisors need to improve with respect to GP training, mainly with respect to general practice concepts (68.0%), training methods (54.8%), and teaching conscientiousness (53.3%). In the process of community practice training, the main aspects that supervisors need to improve are training skills and teaching methods (57.0%), theoretical knowledge and clinical ability (35.4%).

In the subgroup analysis, there were no significant differences in attitudes towards supervisors at different stages (completed training or in training). Further researches are needed to analyze the relationships among the quality of teachers, the expectation of residents with respect to their supervisors and their levels of satisfaction.

### Interview results

GP residents described their experiences with their supervisors. These aspects are described in details in Table 5 with illustrative quotations based on the grounded theory study .

**Table 5:** The categorical relationships formed by the principal axis coding table

**Main coding**	**Submain coding (code frequency)**	**Participants’ initial information**
GP theoretical training supervisors	Supervisors have a solid theoretical foundation and strong teaching conscientiousness (28)	I was satisfied with the lectures. It is hoped that some courses will be offered to help students to cultivate career professionalism . It is suggested that the theoretical courses should not be completed at once. Supervisors are experienced and responsible, and the combination of large classes, small lectures, and group discussions helps a lot.
GP hospital-based training supervisors	GP concept improvement (52)	Residents are more trained as specialists, and they have to tackle some difficult cases. Supervisors in some departments don’t treat patient via the whole-person concept.
	Teaching methods improvement (33)	Supervisors are very willing to teach residents, but their teaching methods are slightly lacking; there is no specific teaching method for GPs; the focus of teaching GPs should be prominent, not just writing history and cases. I hope that supervisors can be trained every two to three months to meet the needs of general practice. Separate teaching will be more effective. I hope supervisors can give students targeted guidance.
	Conscientiousness improvement (51)	Teaching supervisors are quite different, Some supervisors can explain to us the diagnosis and treatment criteria of common and frequently occurring diseases in outpatient clinics and things to which we should pay attention. They can also recommend books for us to go back and see for ourselves, while others just hope that we can help them in their work; their conscientiousness regarding teaching is somewhat weak.
GP community-based training supervisors	Motivation (16)	Community doctors are more responsible. They not only teach various matters that need attention but also provide opportunities for independent medical treatment. At the same time, they also cultivate the awareness of the doctor-patient relationship and help us learn to communicate with patients.
	Teaching methods (34)	Teaching supervisors do not have systematic arrangements; sometimes they don’t have sufficient theoretical knowledge, and the opportunity to practice independently is relatively limited.
	Clinical theoretical knowledge and practice ability improvement (27)	Community-based training supervisors are more responsible, while some of them do not have perfect pedagogy and standard diagnosis and treatment of diseases.

## Discussion

### Summary

GP residents were largely satisfied with their GP theoretical training supervisors. GP hospital-based training supervisors need to improve their conception of GP, teaching methods and teaching conscientiousness. GP community-based training supervisors need to improve their teaching methods, clinical theoretical knowledge and practice ability.

### Reasons and analysis

The government is making great efforts to improve GP training. However, human resource construction with respect to GP supervisors has increased gradually and steadily. GP theoretical training supervisors, GP hospital-based training supervisors and GP community-based training supervisors have different backgrounds and different levels of success with respect to transitioning into their roles as GP supervisors.

Theoretical training supervisors are generally drawn from colleges and are willing to be theoretical training supervisors. During the theoretical training period, GP residents could learn more comprehensive GP concepts, cultivate international visions, and share their opinions smoothly; in addition, supervisors also have opportunities to share their general practice experience with each other. This situation is what affects GP residents’ satisfaction with theoretical training.

GP hospital-based training supervisors are generally drawn from relevant rotations in clinic departments. They are experts in their fields, but they must undertake a difficult transition into the role of GP supervisors, especially with respect to their conception of GP, teaching methods and teaching conscientiousness. The satisfaction of GP residents with GP hospital-based supervisors increased gradually and steadily aside from two periods of decrease during 2008-2010 and 2015-2017, which shows that these supervisors were not trained via a standard model and that the expectations of GP residents may exhibit constant change.

For GP community-based supervisors, the satisfaction of GP residents increased gradually and steadily, although it was relatively low at the early stage. The reality is that community health resources are not effectively used, and the GP community-based training process cannot fully realize its own value. More profound reforms are needed to address this issue.

Moreover, other problems occurred as well. Firstly, management standards for general discipline training are not standardized, and the regulation of management lacks a standardized basis. Secondly, training contents and time allocation need to be further improved. Next, communication and feedback paths are insufficient for residents. Finally, the implementation of relevant support policies is weak, and the incentive system is not perfect with respect to GP teaching.

Compared with this, some of the countries where family medicine is more popular have established their own standards [11–18]. In the UK, to become a general practitioner, one must complete 5-6 years of undergraduate medical education, 2 years of basic training (including clinical rotations in major internal medicine, major surgery, etc.), and 3 years of general practitioner professional training (18 months of comprehensive training and 18 month general practice practice). In the 3-year professional training process, each stage has corresponding requirements (including trainee evaluation, annual appraisal, trainee feedback, competitiveness evaluation, comprehensive evaluation, year-end evaluation, etc.) In the United States, the starting point of vocational training for general practice specialists is the graduates of medical schools (completed a four-year medical school education after graduation), and the training is divided into basic training (3 years) and advanced training (1~2 years). The 3-year basic training includes 2 years of hospital rotation and 1 year of community clinic training. 1-2 years of advanced training can choose a specialty related to general practice as a training program, such as geriatric medicine, rehabilitation medicine, maternal and child health care, tourism medicine, etc. Trained general practitioners must take a unified examination organized by trade associations before they can qualify as specialists in general practice.

At present, in China, although there is training curriculum published by the Chinese Medical Doctor Association in the current time , these is no uniform system among different training bases and across different provinces .The practice of general practitioner training in general hospitals is mainly based on the rotation of hospital department settings, and the community practice mainlyincludes general practice outpatient clinics, establishment of health records, common problems in the community, chronic disease management, focus group population health care, community prevention, etc. Although the training content of general practitioners at home and abroad is basically the same, due to the over-refinement of the specialized settings of domestic general hospitals, the limited business development of community general practitioners, and the lack of general practitioner teachers, the actual training content of general practitioners has become difficult. It is very different from the setting and cannot fully achieve the expected goal. Consequently, there is no uniform standard for GP supervisors in China at present.

GP supervisors training in China is different from those in UK and US in terms of quantity, quality, background, access, supervisor-trainee relationship and assessment methods. The background access and training curriculum are not as strict as those in above countries, and supervisor-trainee relationship is relatively loose. Many Chinese scholars have also discussed whether to formulat a standard for GP supervisors [19–22]. Among them, some suggestions are put forward on the basic concepts of GP supervisors, such as professional quality, clinical medical ability and teaching ability.

The comparison is as Table 6.

**Table 6:** Comparison the GP training program in UK, US and China

Country	Duration	Core competences	Content	Trainer training
UK	10yeas+ ,including 5year undergraduate training , 2 year postgraduate training and 3 year GP Trainee(Hospital & Practice-Based).	Six core competence :Primary Care Management, Community Orientation, Special Problem Solving Capability, Integrated Programs, People-Centered care, Whole-Person Care.	1. Core curriculum statement which provides a full description of the knowledge, skills, attitudes and behaviours required of a GP in managing patients and their problems. 2. Professional, Life Stages, and Clinical topic guides	Undertake an initial induction meeting reviewing the learning needs of the GP trainee and agreeing an educational plan for the post. 2. Complete at least one WPBA(work problem-based assesment ) for the trainee 3. Complete a Clinical Supervisors Report on the Portfolio at the end of the post
US	8~9yeas+ ,including 4 years of undergraduate, 4 years of medical school education, which is divided into basic training (3 years) and advanced training (1~2 years). Advanced training includes specialty related to general practice	Six core competence: Medical Knowledge,Patient Care,Interpersonal & Communication Skills,Professionalism,Practice-Based Learning and Improvement, system-Based Practice,which is trained in different division and circumstances.	The program provide residents with regularly scheduled lectures, conferences, workshops or educational activities. Didactics shall be available for an average of at least five hours per week.	Senior residents or fellows should serve in a supervisory role of junior residents in recognition of their progress toward independence, based on the needs of each patient and the skills of the individual resident or fellow.
China	8 yeas+ ,including 5 year colleage education( simply consepectus class ) 3 year GP residenet training ( including 27 Hospital & 6 months Practice-Based).	No unified and definite stardard,but many research work on it	Theory training: cultivate the concept of GP hospital training: Rotation in different departments in hospital,aiming to disease exposure and community health centre training	56 hours centralized training. Trainees mainly come from general practice , internal medicine, pediatrics, emergency department and other professional directions similar to general practice .

### Policy suggestions

#### Implementing the whole-process tutoring system in the GP resident training program

To solve these issues concerning GP theoretical training supervisors, GP hospital-based training supervisors, and GP community-based training supervisors, the whole-process tutoring [3, 5, 6] system is highly recommended for GP resident training programs. These tutors can be responsible for the whole process of the GP resident training program and can share their experiences with GP supervisors and residents.

The whole-course tutor can provide residents with comprehensive help regarding their needs and can give advices at each stage and facilitate residents’ theoretical study, clinical rotations, and community rotations [1, 4, 7–10].

#### Establishment of a unified standard and screening criteria for supervisors to ensure high-quality supervisors

The quality of teaching staff is an important factor in ensuring the quality of the standardized training of GPs. Specifically, teaching staff quality aims to standardized the qualification of supervisors in each of the training contexts so that senior doctors and associate senior doctors with high degrees of medical ethics, solid theoretical foundations, extensive clinical experience, rigorous clinical thinking, excellent academic standards, good communication skills, and prominent innovative capacity would be selected.

#### Strengthen the training and assessment of supervisors’ teaching ability

Supervisors should be trained regularly with respect to their teaching ability, and various training methods should be adopted to improve their attention to training and enhance their awareness of teaching.

The management department also needs to supervise and inspect the teaching quality of supervisors regularly and to receive feedback from staff. The final evaluation results can serve as an important basis for rewarding and punishing supervisors.

### Study advantages

The study collects a large amount of information concerning the perspectives of general practice residents towards their supervisors and provides a macro-level view of residents’ satisfaction with GP supervisors. A mixed quantitative and qualitative study provides the reader with a comprehensive way to understand and investigate deeper general practice residents’ perspectives towards their supervisors.

### Study limitation

Many factors influence the perspectives of general practice residents towards their supervisors, and the backgrounds of early GP supervisors vary, so further studies regarding other aspects of this topic are needed to find more implications.

## Conclusions

Standardized training of general practitioners by the municipal government and related departments is widely viewed as an important aspect of GP education. General medical education is in line with international conventions, and teams of GP supervisors have been gradually established.

Certain problems also remain with the teaching level of GP supervisors, the standards of general discipline training and the training content emphasised by GP supervisors, etc. This study analyzes the background and perspective of residents towards their supervisors and provides relevant suggestions to strengthen the GP supervisors’ teaching abilities to maintain and improve the quality of GP training.

## Supplementary Information

**Additional file 1.**

## Data Availability

All data and materials are available. The datasets generated for this study are available by contacting the corresponding author.

## Abbreviations

GP: General practice
CHC: Community Health Centre
WPBA: Work problem-based assesment
