# Supplementary material for: Perspectives of general practitioners towards their supervisors over the past ten years in China
Source: BMC Med Educ. 2022 May 31;22:415. doi: 10.1186/s12909-022-03442-3 (PMC9158279; doi:10.1186/s12909-022-03442-3)
Supplement: Supplementary file 1 — Additional file 1. [file 12909_2022_3442_MOESM1_ESM.docx]

|  | Rotation content | Time period (months ) |
| --- | --- | --- |
|  |  |  |
| 1 | theory training （Centralized arrangement or decentralized to various divisions in the whole rotation time ） | 3 |
|  |  |  |
| 2 | clinic rotation training (in hospital's department) |  |
|  | internal medicine | 12 |
|  | neurology | 2 |
|  | pediatric | 2 |
|  | surgical | 2 |
|  | obstetrics and gynecology | 1 |
|  | emergency medicine | 3.5 |
|  | dermatology | 0.5 |
|  | eye | 0.5 |
|  | otorhinolaryngology | 0.5 |
|  | Infectious | 0.5 |
|  | psychiatric | 1 |
|  | Rehabilitation medicine | 0.5 |
|  | traditional Chinese medicine | 0.5 |
|  | optional department | 0.5 |
|  | sub total | 27 |
|  |  |  |
| 3 | practical training in community health center（Interspersed arrangement, total time 6 months ） | 6 |
|  |  |  |
|  | total in three years | 33~36 |

The Rotation content outline of GP resident training in China （2017 version）
